# Cardiotoxicity of BRAF/MEK Inhibitors Mimicking Apical Hypertrophic Cardiomyopathy

**DOI:** 10.7759/cureus.72313

**Published:** 2024-10-24

**Authors:** Jakub Benko, Martin Jozef Péč, Zuzana Tomčová, Monika Péčová, Matej Samoš

**Affiliations:** 1 Department of Internal Medicine, Jessenius Faculty of Medicine, Martin, SVK; 2 Department of Cardiology, Faculty Hospital Nitra, Nitra, SVK; 3 Department of Oncology, Faculty Hospital Nitra, Nitra, SVK; 4 Department of Oncology, Jessenius Faculty of Medicine, Martin, SVK

**Keywords:** cardio-oncology, ctrcd, left ventricular strain, melanoma, ras inhibition, targeted therapy

## Abstract

Cardio-oncology is a new and fast-evolving collaborative subdiscipline of cardiology whose goal is to increase the quality and length of the lives of oncological patients with precise prophylactic and therapeutical interventions. Novel targeted therapies present a challenge to recognize and treat rare adverse cardiovascular effects, usually without any evidence-based guidance. Therefore, scrupulous descriptions of clinical cases and drafting trials for real-world safety are paramount. We present a case of a 50-year-old Caucasian female who was diagnosed with cancer therapy-related cardiac dysfunction caused by combined BRAF and MEK inhibition in the treatment of malignant melanoma. The patient presented atypically with isolated severe fatigue without other specific cardiovascular symptoms. The findings of echocardiography and cardiac magnetic resonance indicated apical hypertrophic cardiomyopathy (AHCM), which is rare in our region. We stopped the oncological therapy and treated the patient with an ACE inhibitor, a beta-blocker, and an SGLT-2 inhibitor with a satisfactory effect. During the upcoming six months, the patient’s state improved, and the control echocardiography ruled out an AHCM. As the patient’s state rapidly improved after the withdrawal of BRAF and MEK inhibitors and the morphological finding was reversible, it can be theorized that the cardiotoxicity caused by the aforementioned drugs mimicked the phenotype of AHCM.

## Introduction

Targeted oncological treatment significantly improved many oncological patients' prognosis and quality of life. Inhibitors of the RAS cascade and immune checkpoint inhibitors form the cornerstone of adjuvant therapy for malignant melanoma [1]. Both therapies can cause significant cardiovascular adverse events. BRAF and MEK inhibition are connected to left ventricular systolic dysfunction and heart failure, with a higher incidence when used in combination therapy. The incidence ranges in different studies from 2% to 12% and is usually reversible after medication withdrawal [2]. On the other hand, immune checkpoint inhibitors can cause myocarditis even with fatal outcomes [3]. Apical hypertrophic cardiomyopathy (AHCM) is a form of hypertrophic cardiomyopathy (HCM). It affects men more commonly than women and is more prevalent among Asian ethnicities, representing up to 40% of HCM cases. The prevalence is much lower in Europe, constituting just 8% of all cases. The typical diagnostic findings are an increased thickness of more than 15 mm of the left ventricle apex found on echocardiography or cardiac magnetic resonance and giant negative T waves on the ECG [4].

## Case presentation

A 50-year-old female patient without known cardiorespiratory diseases was sent to our cardio-oncology clinic for cardiovascular assessment because of fatigue and tachycardia. The patient was treated for BRAF-mutated malignant melanoma of the temporoparietal head region with metastasis of adjacent skin and neck lymph nodes solved surgically by radical dissection. Adjuvant therapy with dabrafenib and trametinib was introduced. Our examination took place nine months after the start of the adjuvant therapy. The patient complained of severe progressive fatigue evolving during the last three months and was unable to walk more than 10 meters without resting due to muscle weakness with marked functional limitation. She stated no chest pain or dyspnea and was without other complaints. Her family history was negative for early cardiovascular diseases, cardiomyopathies, and sudden cardiac death. Her vitals were normotension without orthostatic hypotension, tachycardia of 120 bpm, resting saturation of 97%, and her BMI was 34 kg/m^2^. No signs of systemic congestion were present, and the heart auscultation was normal.

**Figure 1:**
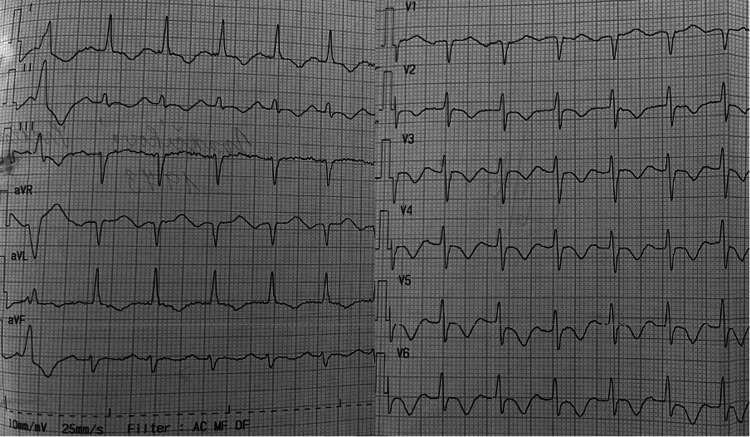
The 12-lead ECG showing diffuse negative T waves suggesting hypertrophic cardiomyopathy

The initial ECG showed sinus tachycardia and diffuse repolarization changes throughout all the leads (Figure 1). D-dimer was positive, and a CT pulmonary angiogram was performed without findings of pulmonary embolism or lung congestion. The concentration of high-sensitivity troponin T was mildly elevated (42 ng/l, reference value < 12 ng/l), and NT-proBNP was markedly increased (9767 pg/ml, reference value < 125 pg/ml). Other laboratory findings were normal (potassium 4 mmol/l, reference values 3.4-5.2; magnesium 0.8 mmol/l, reference values 0.77-1.03 mmol/l; creatinine 77 μmol/l, reference values 44-104 μmol/l; total bilirubin 12.5 μmol/l, reference values 5-28 μmol/l; ALT 0.6 μkat/l, reference value < 0.85 μkat/l).

**Figure 2:**
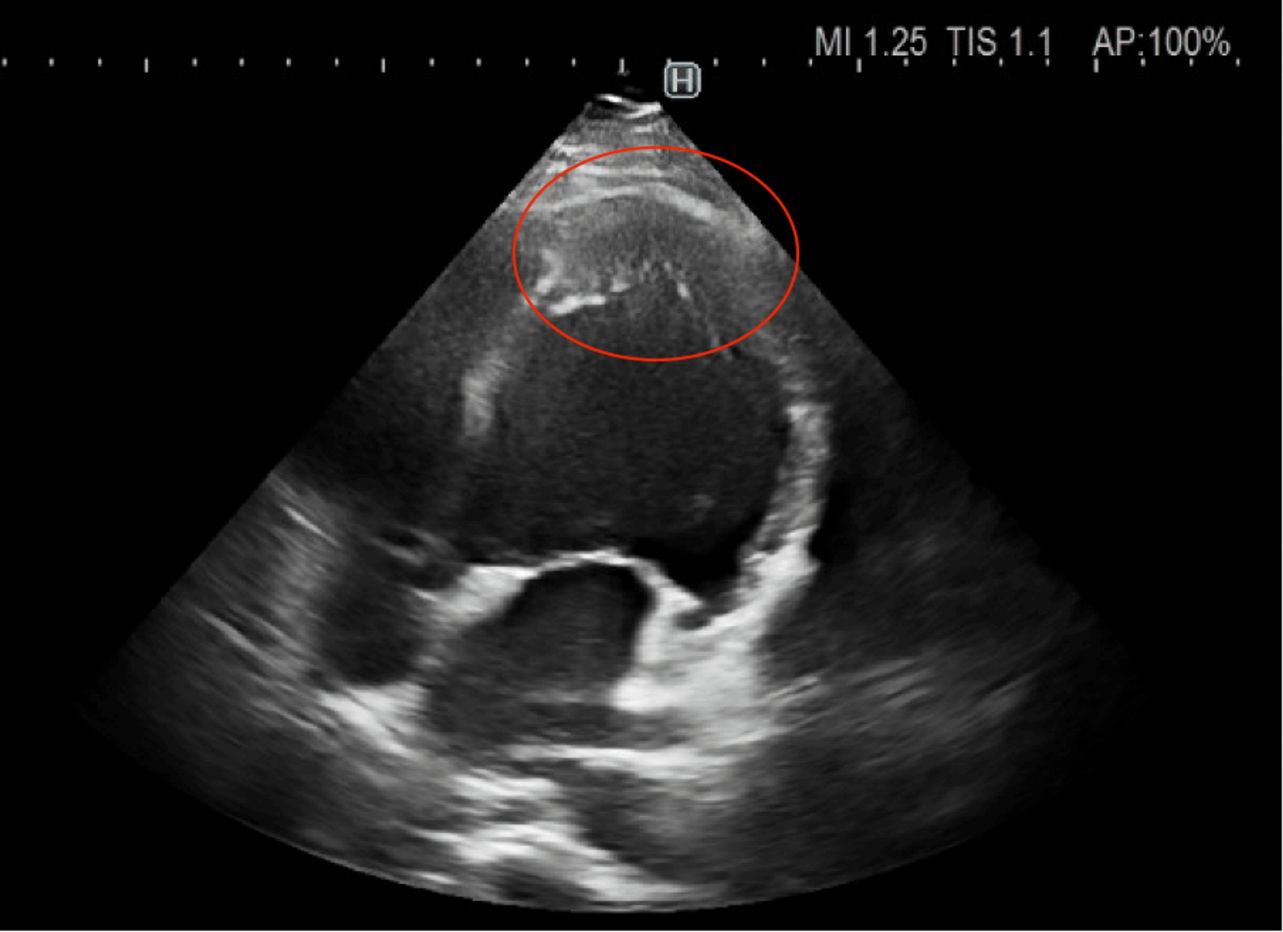
Apical four-chamber view showing a thickened apex of the left ventricle

**Figure 3:**
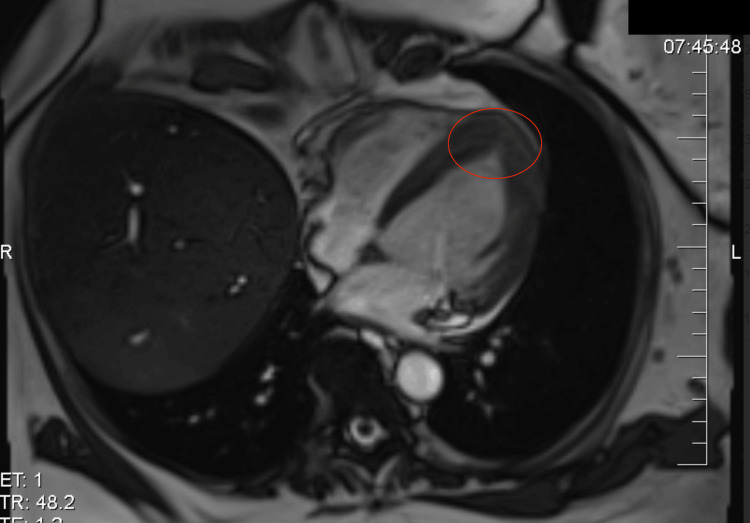
The cardiac magnetic resonance showing an ace-of-spade sign indicating apical hypertrophic cardiomyopathy

**Figure 4:**
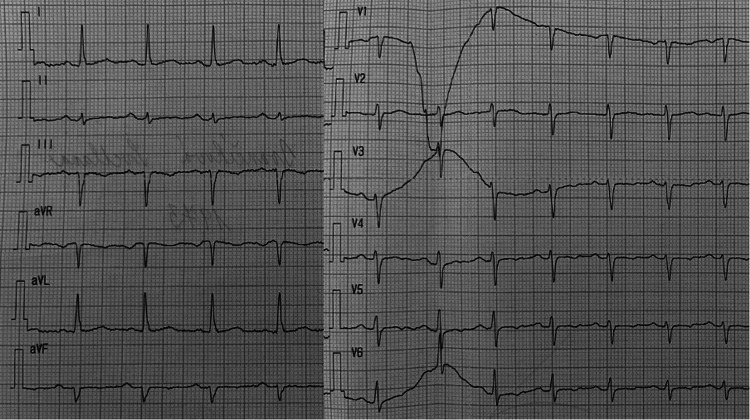
The ECG showing improvement of repolarisation changes after withholding anticancer therapy and appropriate treatment

**Figure 5:**
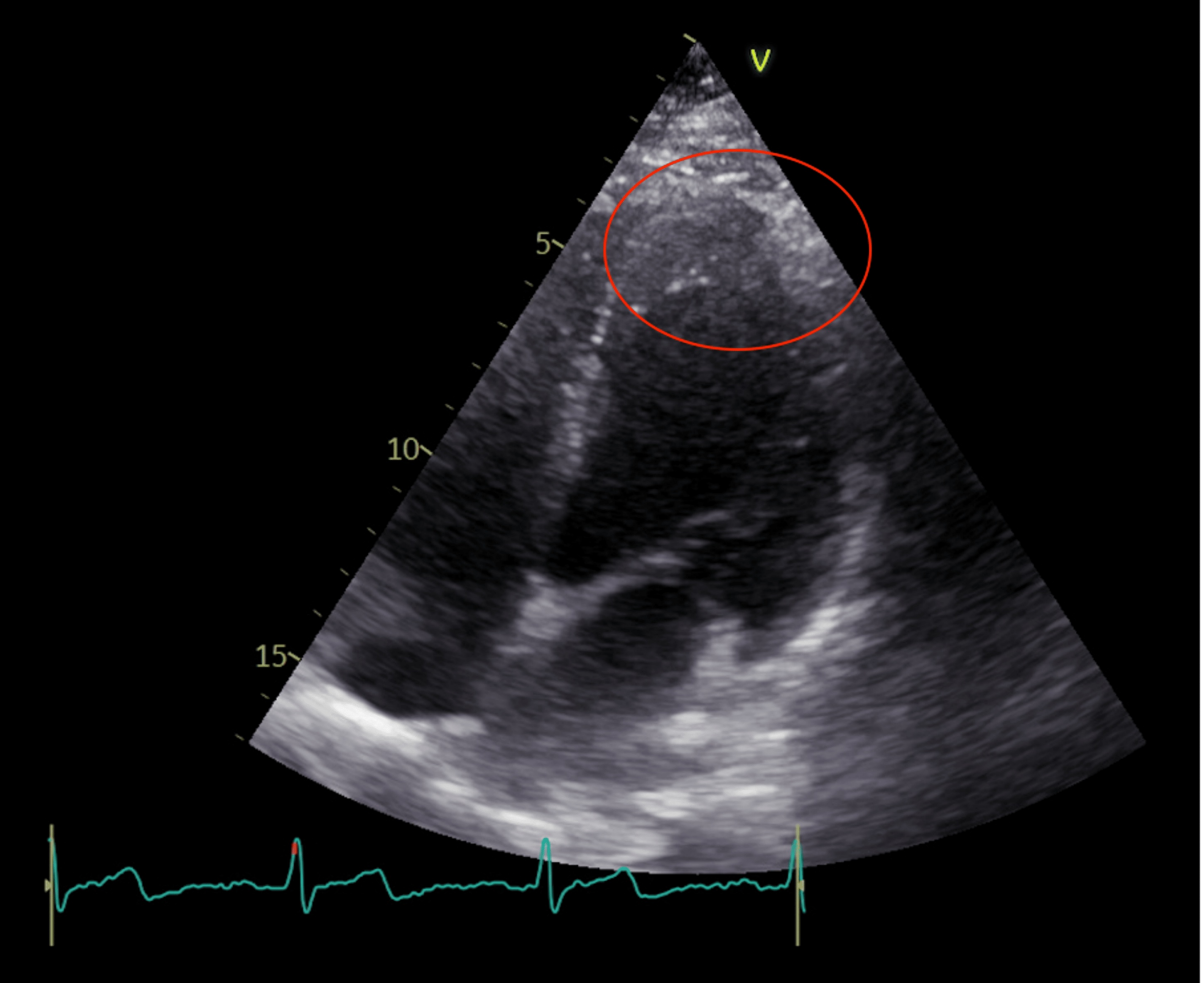
The apical four-chamber view showing increased thickness (12 mm) of the apex of the left ventricle

**Figure 6:**
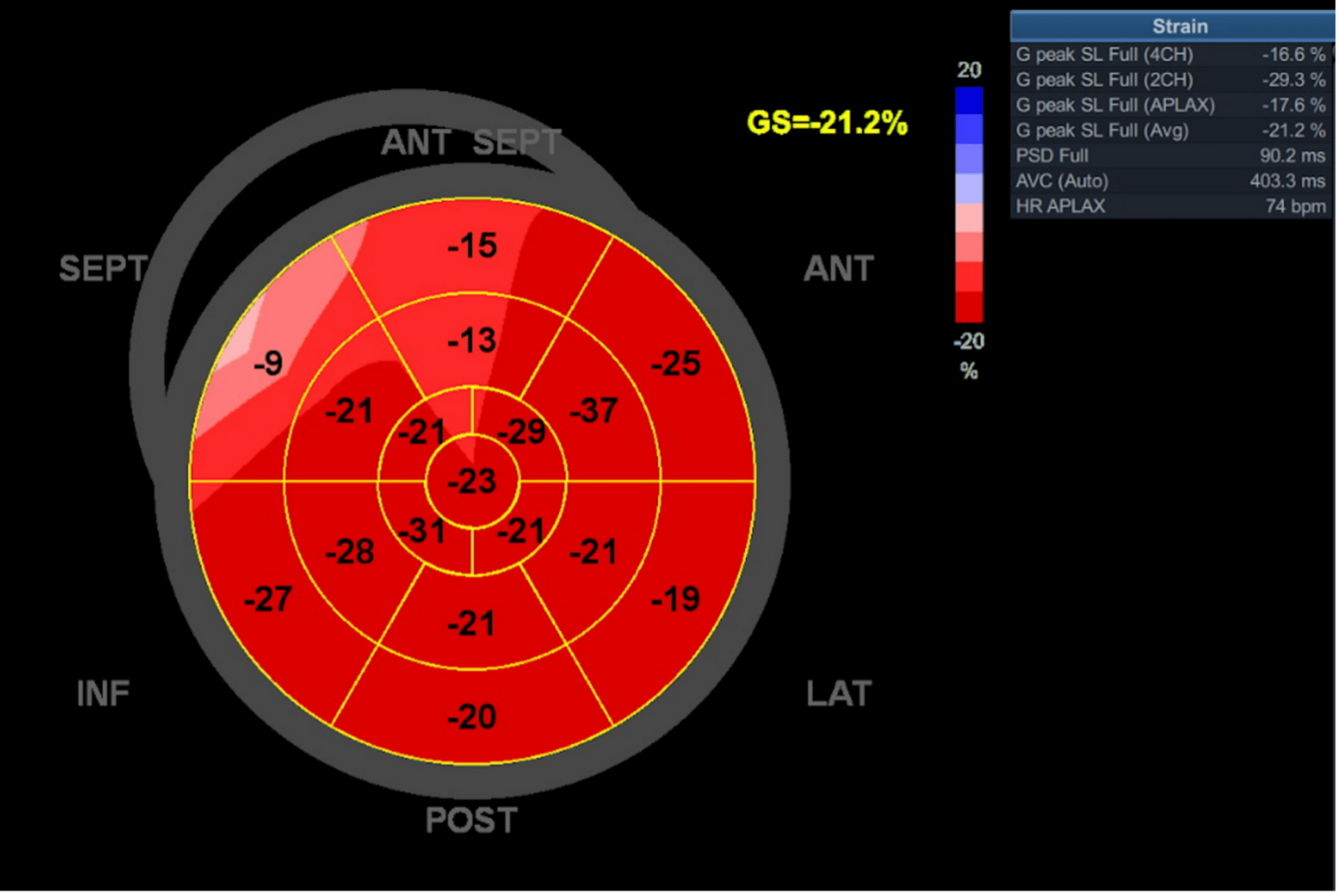
Left ventricular strain measurement after the treatment

The transthoracic echocardiography showed a thickened apex of the left ventricle with a preserved ejection fraction of the left ventricle without regional wall motion abnormalities. There were no echocardiographic signs of takotsubo syndrome or specific findings of pulmonary hypertension (Figure 2). We could not examine the left ventricle's strain parameters at that time because of the patient's insufficient echogenicity and technical issues. To exclude coronary artery disease, a CT coronary angiography was performed without findings of coronary stenosis, and the Agatston score was zero. Cardiac magnetic resonance showed a preserved ejection fraction of the left ventricle (51%), but a diffuse reduction of all left ventricular strain parameters (global radial strain 9.3%, global circumferential strain 7.0%, global longitudinal strain 8.9%), and hypertrophy of the apical region of the left ventricle without any signs of myocarditis. The left ventricular outflow tract was without signs of obstruction, and the gradient was low. A case of AHCM was suspected (Figure 3). An SGLT-2 inhibitor, beta-blocker, and ACE inhibitor were introduced, and the oncological therapy was stopped. After one month, the patient’s state improved significantly and had only slight functional limitations. The concentration of troponin (29 ng/l) and NT-proBNP (426 pg/ml) dropped. The repolarization changes on ECG were less pronounced (Figure 4). We repeated the echocardiography after six months with normal global longitudinal strain and thickening of the left ventricular apex not meeting the criteria of AHCM (Figures 5, 6). Also, all the other walls of the left ventricle decreased in thickness. Her cardiac laboratory markers were normalized. Because of melanoma relapse, the patient was started on pembrolizumab six months later. The patient is still being followed up on at our cardio-oncology clinic regularly.

## Discussion

Combined BRAF and MEK inhibition can typically cause left ventricular dysfunction, confirmed by decreased ejection fraction of the left ventricle or left ventricular strain when available. The incidence from clinical studies is 2% to 12% but can be underestimated. The RAS-RAF-MEK-ERK signaling pathway, which is modulated by BRAF and MEK inhibitors, plays an important role in remodeling the heart and is the reason for cardiotoxicity development [2]. However, the development of HCM was not associated with the usage of these drugs before. When signs of heart failure develop, standard treatment according to the newest guidelines and cardioprotection with an inhibitor of the renin-angiotensin system is indicated [5].

According to the latest focused update of the European Society of Cardiology, the mainstay of therapy for heart failure with preserved ejection fraction is an SGLT-2 inhibitor. Loop diuretics are used when signs of fluid retention are present [6]. The mainstay of treatment for HCM is betablockade [4]. That is why we chose to treat the patient with a combination of an SGLT-2 inhibitor, a beta-blocker, and an ACE inhibitor.

As a previous echocardiography was not available, we suspected a concomitant AHCM and cancer therapy-related cardiac dysfunction (CTRCD), even though the prevalence of AHCM in Central Europe is low. Another factor that does not point in the direction of AHCM is a negative family history. With a fast improvement in the ECG changes and morphological findings after anticancer therapy withdrawal, just a CTRCD without AHCM is much more likely, as CTRCD associated with BRAF and MEK inhibitors and also other tyrosine kinase inhibitors is often reversible [7]. We were unable to classify the risk of BRAF and MEK cardiotoxicity according to the Heart Failure Association (HFA)-International Cardio-Oncology Society (ICOS) tool as the patient did not have echocardiography or cardiac markers examined before the start of treatment of the melanoma. The score uses a multifactorial approach to establish the potential risk of cardiotoxicity of different anticancer therapies. It considers the patient's previous cardiovascular diseases, cardiac imaging and biomarkers, age, cardiovascular risk factors, current and previous cancer treatment, and lifestyle risk factors [5]. According to the study of Glen et al., cardiotoxicity commonly affects patients deemed low or moderate-risk and therefore suggests rigorous echocardiographic monitoring during the treatment regardless of the risk status [7].

## Conclusions

We present a case report which highlights the cardiotoxicity of combined BRAF and MEK inhibition. The patient developed heart failure with preserved ejection fraction and significant functional limitation morphologically mimicking apical hypertrophic cardiomyopathy. The patient's state improved rapidly after stopping the anticancer therapy and initiating standard heart failure medication. The control echocardiography showed a significant improvement in the left ventricular strain, which confirms the etiology. Our case also highlights the importance of left ventricular strain examination, as this can also clarify more subtle echocardiographic dysfunction of the left ventricle. Finally, it can be theorized that combined BRAF and MEK inhibition can cause a morphological picture of an apical type of hypertrophic cardiomyopathy in a subgroup of treated patients.
